# Long-term outcome of the vaginoscopic approach for infantile vaginal yolk sac tumors: A case report

**DOI:** 10.1016/j.ijscr.2024.110730

**Published:** 2024-12-10

**Authors:** Fang Li, Jing Chen, Yu Zhang, Lang Pang, Dingyuan Zeng

**Affiliations:** a Department of Gynecology, Jincheng Hospital Affiliated to Changzhi Medical College, Jincheng People's Hospital, Shanxi, China; b Center for Reproductive Medicine, Guangzhou Women and Children's Medical Center Liuzhou Hospital, Guangxi, China; c Department of Gynecology, Guangzhou Women and Children's Medical Center Liuzhou Hospital, Guangxi, China; d State Key Laboratory of Ultrasound in Medicine and Engineering, College of Biomedical Engineering, Chongqing Medical University, Chongqing, China; e Department of science and education, Guangzhou Women and Children's Medical Center Liuzhou Hospital, Guangxi, China; f Department of Pathology, Guangzhou Women and Children's Medical Center Liuzhou Hospital, Guangxi, China

**Keywords:** Infantile vaginal yolk sac tumor, Vaginoscopic approach, Fertility preservation, Case report

## Abstract

**Introduction:**

Infantile vaginal yolk sac tumor (YST) is a rare and aggressive form of pediatric cancer that often presents with bloody discharge. Despite advances in chemotherapy, managing post-chemotherapy AFP level rebounds remains a challenge. This case report describes a 7-month-old girl with vaginal YST whose AFP levels rose following 3 cycles of PEB chemotherapy.

**Case presentation:**

Despite aggressive PEB chemotherapy, the patient's AFP levels increased, indicating ongoing disease activity. MRI scans failed to reveal any visible lesions, suggesting a potential oversight in disease assessment. However, a vaginoscopy uncovered a 1.5-cm residual lesion in the vaginal area, highlighting the importance of this procedure in identifying occult disease. The lesion was successfully resected, and histopathological examination confirmed clear margins. Postoperatively, there was a marked decrease in serum AFP levels, and no tumor recurrence was observed over a 60-month follow-up period. The patient's ovarian function and uterine development were preserved.

**Clinical discussion:**

This case underscores the value of a multimodal approach in managing vaginal YSTs, particularly when standard imaging fails to detect residual disease. The vaginoscopic approach not only identified a residual lesion missed by MRI but also allowed for minimally invasive treatment, reducing the need for additional chemotherapy and its associated side effects.

**Conclusion:**

The vaginoscopic approach provides a significant alternative for treating vaginal YSTs, especially in cases where AFP levels rise post-chemotherapy. This method emphasizes fertility preservation and minimizes the impact on reproductive health, offering a promising direction for future treatment strategies in pediatric oncology.

## Highlights

• Vaginoscopic resection offers a new approach for vaginal YSTs with post-chemotherapy AFP rebound.
• ‘No touch’ technique preserves hymenal integrity, reduces cervical complications.
• Vaginoscopy identified residual disease despite negative MRI.
• Less invasive approach preserves fertility, with no recurrence in 60 months.
• Emphasizes fertility preservation in infantile YST treatment, calls for further research.

## 1 Introduction

Germ cell tumors (GCTs) in children are relatively rare, accounting for 1 % to 3 % of all pediatric tumors [1]; vaginal GCTs make up 3 % to 8 % of all GCTs; and vaginal yolk sac tumors, as a subtype of vaginal GCTs, have an even lower incidence rate [2,3]. The treatment of infantile vaginal yolk sac tumor (YST) represents a significant milestone in the field of pediatric medical oncology. This rare and aggressive neoplasm primarily affects girls under the age of 3, often manifesting as symptoms such as bloody vaginal discharge [4]. Elevated serum alpha-fetoprotein (AFP) is a common hallmark, that serves as a diagnostic, therapeutic, and prognostic indicator [5,6]. Imaging modalities such as ultrasound, computed tomography (CT) and magnetic resonance imaging (MRI) play a supportive role in diagnosis, while pathological examination remains the gold standard for confirming YSTs [7]. The combination of imaging features and histopathological examination allows for the exclusion of other diseases that can cause increased levels of alpha-fetoprotein (AFP), such as hepatoblastoma, teratomas containing yolk sac elements, and other germ cell tumors. Over time, there has been a significant shift in the treatment paradigm for vaginal yolk sac tumors. Initially, more aggressive interventions were favored, including chemotherapy combined with surgical procedures such as partial vaginal resection along with partial cystectomy or even radical hysterectomy [8,9]. However, this approach has since transitioned toward more conservative strategies, with chemotherapy now serving as the primary mode of treatment [2,10,11]. This conventional approach is rooted in the understanding of the disease's chemosensitivity and the desire to remove any identifiable tumor burden [12,13].

The PEB regimen, which consists of cisplatin, etoposide, and bleomycin, is currently the most widely utilized chemotherapy protocol for the treatment of vaginal YSTs [5,14]. The PEB chemotherapy should be administered continually until patients achieve complete biochemical remission, as indicated by normalization of the serum AFP level [15]. In a review spanning the past three decades by Bhatt et al. [16], recurrence rates following chemotherapy alone versus surgery combined with chemotherapy were found to be 14 % and 13 %, respectively, with no significant variance.

A significant challenge in the management of vaginal YSTs is addressing the rebound of the AFP level following chemotherapy. This case exemplifies the challenge of managing post-chemotherapy AFP rebounds, prompting the introduction of our proposed solution.

## 2 Methods

The study collected a case of vaginal YST in a child. This study has adhered to the SCARE guidelines in its reporting [17]. Written informed consent for publication was obtained from the patient's guardians before proceeding with the publication of this case report.

## 3 Case presentation

**Fig. 1:**
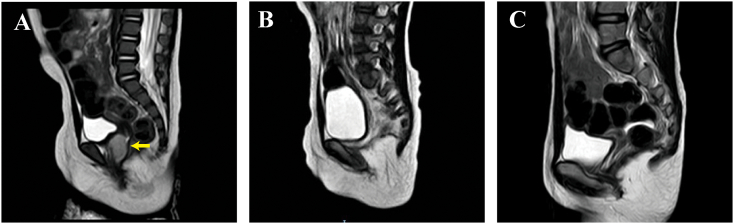
Sagittal MRI of the pelvis before and after chemotherapy. A) Before chemotherapy, a round and well-defined vaginal mass (arrow) was observed. B) After 3 cycles of chemotherapy, no significant vaginal tumor was detected. C) Follow-up MRI at 60 months revealed no evidence of a mass in the anterior vagina.

**Fig. 2:**
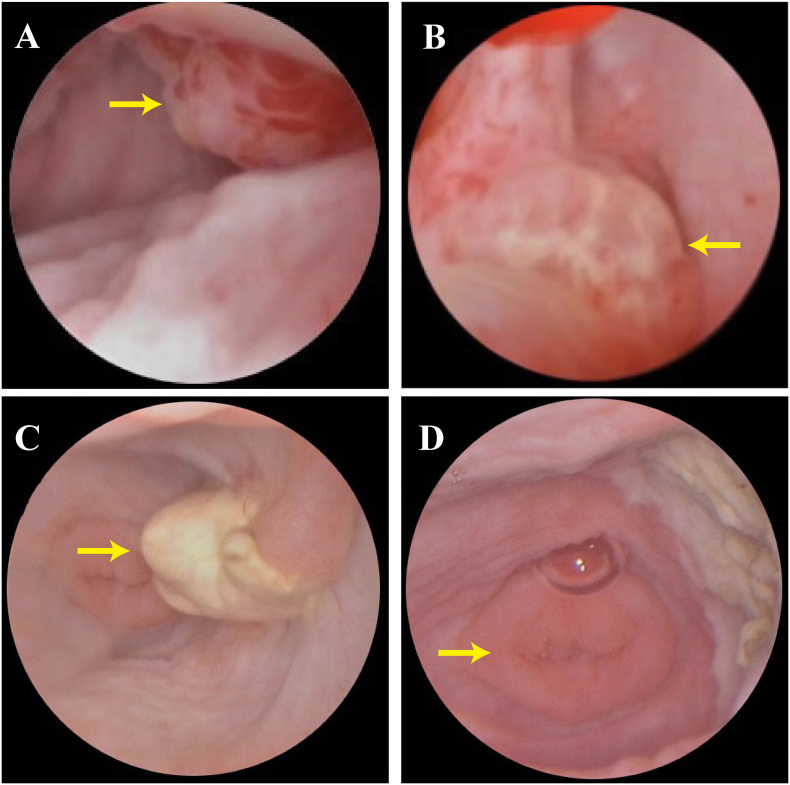
Vaginoscopy before and after chemotherapy. A & B) Vaginal mass before chemotherapy: a red and white botryoid mass (arrow) was visible on in the anterior of the vagina. C) A white and atrophic mass residue (arrow) was observed after chemotherapy. D) Vaginoscopic view after removal of the mass by vaginoscopic operation, showing a normal cervix (arrow). (For interpretation of the references to colour in this figure legend, the reader is referred to the web version of this article.)

**Fig. 3:**
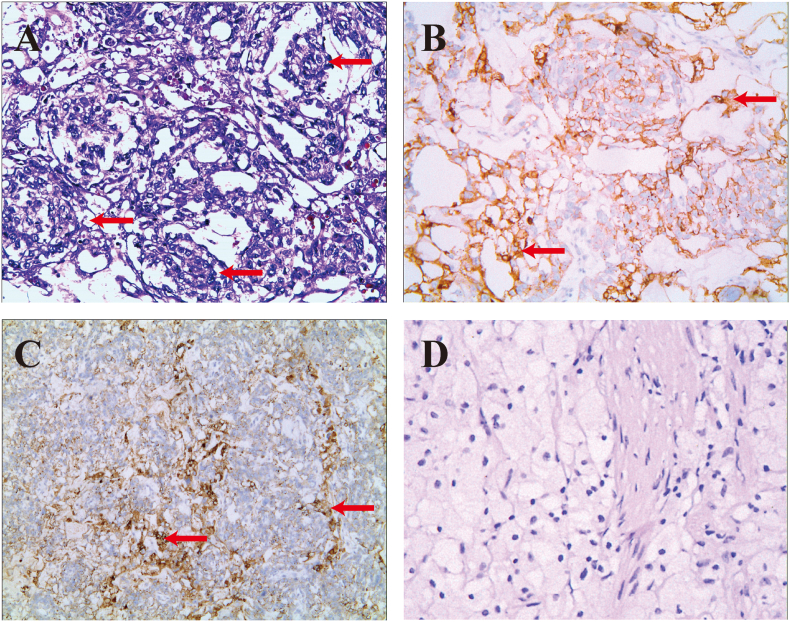
Histological and immunohistochemical examination before and after chemotherapy. Eosinophilic Periodic acid-Schiff stained section of yolk sac tumors showed a reticular pattern with occasional Schiller-Duval bodies (arrow) before (A, ×100). The tumor cells were diffusely positive for Glypican-3 (B, ×100) and Ki-67 (C, ×100) but not after chemotherapy (B, ×100). With H&E staining, Schiller-Duval bodies were not observed after chemotherapy (D, ×100).

In June 2018, a seven-month-old girl was admitted to our gynecology department with a three-hour history of vaginal bleeding and a piece of tissue measuring 2.5 cm × 1 cm × 1 cm in her diaper. MRI revealed a heterogeneous mass measuring 1.5 cm × 1.2 cm × 2.2 cm occupying the vagina (Fig. 1A). Her serum AFP was highly elevated at 5623.81 ng/mL, while her β-HCG levels remained within normal limits. Hysteroscopy revealed a large friable vaginal mass with a “cluster of grapes” appearance in the anterior vagina. (Fig. 2A & B). No metastatic lesions were found, and pathology confirmed YST. The diagnosis was substantiated by both Hematoxylin and Eosin (H&E) staining and immunohistochemical staining for Glypican-3 and Ki-67 (Fig. 3A & B & C). Glypican-3 and Ki-67 stainings highlight the cellular characteristics of the tumor, assisting in confirming the diagnosis of YST.

Treatment involved BEP administered every 3 weeks. After 3 cycles of chemotherapy, her serum AFP level decreased to 15.22 ng/mL. During this period, the patient only exhibited a decrease in appetite and did not experience any other adverse reactions. However, one week after discontinuation of chemotherapy, her AFP level rebounded to 22.38 ng/mL, with persistent vaginal fluid. Despite MRI showed no mass (Fig. 1B), a vaginoscopy revealed a 1 × 0.5 × 0.5 cm white mass (Fig. 2C). Local surgical excision of the vaginal mass was performed, and pathology demonstrated polypoid tissue and showing no evidence of abnormal mitotic figures in the tumor cells (Fig. 3D). Postoperative serum AFP level decreased to 7 ng/mL, indicating effective tumor clearance. Based on the Children's Oncology Group (COG) staging criteria for pediatric germ cell tumors [18], and considering the surgical grading performed, the tumor was classified as stage I.

Subsequent surveillance vaginoscopy at 6 months post-tumor resection indicated normal vaginal mucosa with a total vaginal length of 4.5 cm, and visible normal cervical tissue of a prepubertal girl (Fig. 2D). At the 60-month follow-up, no recurrence was observed, and the child's ovarian function and uterine development were found to be normal. Hormonal assays revealed an anti-Müllerian hormone (AMH) level of 8.11 ng/mL, an estradiol (E2) level below the threshold of 10 pg/mL, a follicle-stimulating hormone (FSH) level of 1.12 mIU/mL, and a luteinizing hormone (LH) level of 0.03 mIU/mL, all of which are within the normal range for her age. Additionally, serum alpha-fetoprotein (AFP) levels were within the normal range, and magnetic resonance imaging (MRI) showed no signs of recurrence (Fig. 1C).

## 4 Discussion

This case report details the management of vaginal YSTs through a novel approach involving vaginoscopic resection. In our case, the persistent increase in AFP levels after chemotherapy led us to propose a comprehensive treatment strategy involving chemotherapy, vaginoscopic resection, and subsequent chemotherapy. This vaginoscopic approach, utilizing a ‘no touch’ hysteroscopic technique, aims to reduce the long-term impact on sexual and reproductive functions by minimizing the number of chemotherapy sessions and the associated side effects. The procedure is conducted through a transvaginal approach without the use of a speculum to dilate the vaginal introitus, thus preserving the integrity of the hymen. Additionally, by avoiding the use of cervical forceps, this method reduces the invasiveness to the cervix, thereby decreasing the likelihood of complications such as vagal nerve stimulation syndrome [19].

The challenge of managing a rebound in serum AFP levels after three chemotherapy cycles in our case highlights the complexities of YST treatment. Traditional salvage interventions, such as altering chemotherapy regimens or radical surgery, carry significant risks to the patients ‘long-term quality of life and reproductive health [20,21]. The PEB chemotherapy regimen can lead to renal dysfunction, pulmonary fibrosis and neurotoxicity [21]. Neurotoxicity encompasses a range of symptoms such as ototoxicity, which can lead to hearing impairment, sensory loss, and in severe cases, peripheral paresthesia or paralysis [22]. These considerations necessitate a cautious and minimal approach to the number of chemotherapy cycles administered, particularly in the infants and young children. From the current literature, the PEB chemotherapy regimen is considered to have the least side effects and is currently seen as the most effective chemotherapy protocol. However, due to the rarity of cases, there is a lack of large-sample statistical data, and the evidence for the effectiveness of chemotherapy alone remains limited. The use of vaginoscopic surgery allows for early intervention, which on one hand can confirm the diagnosis and rule out mixed germ cell tumors, and on the other hand, in cases where chemotherapy is not effective and AFP rebounds, the intervention of vaginoscopy and complete resection of the lesion can significantly reduce postoperative AFP, also providing ideas and opportunities for subsequent treatment. Whether it can reduce the number of chemotherapy sessions still requires further large-sample research data to substantiate this claim.

Despite MRI findings suggesting no visible vaginal lesions, the presence of abnormal AFP levels and vaginal discharge indicated ongoing disease activity. Vaginoscopy was crucial for identifying residual lesions not apparent on MRI, which is attributed to the MRI's limitation in differentiating between treatment-induced changes and actual tumor signals, as well as its inability to detect small residual lesions that may be present within dense fibrotic tissue. Complete excision for pathological assessment was essential to rule out mixed germ cell tumors and inform the subsequent treatment plan, leading to a decrease in AFP levels and symptom alleviation.

The vaginoscopy marks a significant shift in YST management, offering a less invasive alternative to traditional treatments. This technique preserved the patient's fertility and reproductive capabilities, underscoring the importance of considering long-term quality of life in treatment decisions. The complete resection of residual lesions and the reduction in chemotherapy cycles demonstrated sustained efficacy, with no tumor recurrence occurring after 60 months, highlighting the effectiveness of this method.

The successful outcome of this case illustrates the potential of the vaginoscopy as a valuable alternative for treating infantile vaginal YSTs, particularly in patients in whom AFP levels do not decrease with standard chemotherapy. This technique has the potential to extend beyond the treatment of vaginal YSTs. In the pediatric patient, it can be applied to other GCTs and rhabdomyosarcomas, where fertility preservation is crucial and minimizing the impact on sexual and reproductive functions is essential. Additionally, its use in managing early cervical, vaginal lesions, foreign bodies in the vagina, and other abnormal uterine bleeding underscores its versatility. The minimally invasive nature of this technology, combined with its ability to maintain anatomical integrity, makes it an attractive option for a broad range of clinical applications. Future research should focus on validating this technique in various malignant tumors and developing standardized protocols to maximize its applicability. Concurrently, it is necessary to validate the efficacy and safety of the vaginoscopy in a large multicenter cohort, with the aim of improving outcomes, enhancing quality of life, and preserving fertility in children with rare tumor diseases, such as infantile vaginal YSTs.

## 5 Conclusions

The vaginoscopy for the resection of YSTs offers a significant advantage for patients who exhibit an increased in AFP levels during chemotherapy. It enables noninvasive removal of residual lesions, preserving vaginal integrity and obviating the need for modifications in chemotherapy regimens or more radical surgeries such as hysterectomy. Such interventions can prevent the loss of sexual and reproductive function, aiming to preserve fertility and enhance the quality of life for affected children in the future.

## Author contribution

LF conceptualized the study design. LF and CJ prepared the initial draft of the case report. LP conducted the histological analysis and prepared the tissue sections. CJ and ZY were responsible for the preparation of the figs. LF, CJ, ZY, LP, and ZDY critically reviewed the manuscript data and contributed to the final draft of the manuscript. All authors have reviewed and approved the final manuscript.

## Consent for publication

Written informed consent for publication was obtained from the patient's guardians before proceeding with the publication of this case report. Upon request by the Editor-in-Chief, a copy of the signed consent form will be provided for editorial review.

## Ethical approval

This study was conducted with the approval of an ethics committee (approval number KS-KY-2024-208). All the data were collected in accordance with the principles of the Declaration of Helsinki.

## Guarantor

Mrs. Fang Li.

## Research registration number

Not applicable.

## Funding

This work was supported by the Guangxi Science and Technology Plan Project (Guangxi Clinical Research Center for Obstetrics and Gynecology, GuiKeAD22035223). The role of the funding body was purely financial, with no influence on the study design, data collection, analysis, interpretation, or writing of the report.

## Conflict of interest statement

The authors declare that they have no competing interests.
